# Risk of recurrent venous thromboembolism and major bleeding according to risk factor profiles in Asian patients: a subgroup analysis EINSTEIN-Extension and EINSTEIN-CHOICE

**DOI:** 10.1186/s12959-024-00609-4

**Published:** 2024-06-06

**Authors:** Norikazu Yamada, Weiguo Fu, Zhenyu Shi, Ki-Hyuk Park, Hyo-Soo Kim, Xiangchen Dai, Anthonie WA Lensing, Akos F Pap, Tomoko Kohno, Tsubasa Tajima, Tadashi Watakabe, Tomoyuki Mitsumori

**Affiliations:** 1 Kuwana City Medical Center, Kotobuki-Cho 3- 11, 511-0061 Kuwana City, Mie Japan; 2 Departments of Vascular Surgery, Zhongshan Hospital, Fudan University, Shanghai, China; 3 Division of Vascular and Endovascular Surgery, Department of Surgery, Daegu Catholic University School of Medicine, Daegu, Korea; 4 Department of Cardiology, Seoul National University Hospital, Seoul, South Korea; 5 Department of Vascular Surgery, Tianjin Medical University General Hospital, Tianjin, China; 6 Bayer AG, Wuppertal, Germany; 7 Bayer Yakuhin Ltd, Osaka, Japan

**Keywords:** Aspirin, Rivaroxaban, Venous thromboembolism, Bleeding, Anticoagulation

## Abstract

**Background:**

Risks of recurrence and major bleeding with extended anticoagulation in Asian patients with venous thromboembolism (VTE) are similar to those in non-Asian patients but risks according to baseline risk factor profiles is not well documented.

**Methods:**

Subgroup analysis of two randomized trials, which compared once-daily rivaroxaban (20 mg or 10 mg) with placebo or aspirin (100 mg) for extended treatment in Asian patients with VTE who had completed 6–12 months of anticoagulation. Index events were classified as unprovoked, provoked by major persistent risk factors, minor persistent risk factors, minor transient risk factors, or major transient risk factors. One-year cumulative risks of recurrent VTE were calculated for these risk factor profiles.

**Results:**

367 patients received rivaroxaban, 159 aspirin, and 48 placebo. For patients with unprovoked VTE, one-year cumulative incidences of recurrence in the 202 patients given rivaroxaban, the 89 given aspirin and the 28 given placebo were 1.6%, 5.8%, and 14.8%, respectively. For patients with VTE provoked by minor persistent risk factors, these incidences were 0% in the 74 patients given rivaroxaban, 9.3% in the 36 given aspirin, and 0% in the 12 given placebo. No recurrent VTE occurred in patients with VTE provoked by major persistent or transient risk factors or minor transient risk factors. Rivaroxaban was not associated with a significant increase in major bleeding.

**Conclusions:**

Rivaroxaban seems to be an effective and safe option for extended treatment in Asian patients, especially those presenting with unprovoked VTE. Subgroups of patients with provoked risk factors were too small to draw meaningful conclusions.

**Trial registration:**

NCT00439725 and NCT02064439.

## Introduction

Current guidelines on antithrombotic treatment in patients with symptomatic VTE recommend that the decision to extend treatment beyond 3 months be based on the balance between the risk of recurrent VTE if treatment is stopped and the risk of bleeding with continued treatment [1, 2], taking patient preferences into account. A duration of anticoagulant treatment of 3 to 6 months is recommended for patients with VTE provoked by major transient risk factors [1, 2], whereas continuation of anticoagulation beyond 3 to 6 months is suggested for patients with VTE that is unprovoked or related to major persistent risk factors unless the risk of bleeding is high [1, 2]. 

Recently, a prespecified analysis of 2 randomized trials [3–5], which compared once-daily rivaroxaban (20 mg or 10 mg) with aspirin (100 mg) or placebo for extended VTE treatment, confirmed the high risk of recurrent VTE following cessation of anticoagulant therapy in patients with unprovoked VTE or VTE provoked by a major persistent risk factor. It also confirmed the low risk if anticoagulation is stopped in patients with VTE provoked by a major transient risk factor. However, recurrence rates in patients with VTE provoked by minor persistent or minor transient risk factors were not significantly lower than those in patients with unprovoked VTE, whereas rivaroxaban reduced the risk of recurrent VTE by > 75% compared with placebo. The results supported regulatory approval of a rivaroxaban label update, providing physicians and patients flexibility to choose reduced dose rivaroxaban for extended secondary prevention of VTE.

In a recent global, prospective, non-interventional study of real-world treatment practices [6], Asian patients with VTE were less likely than patients from the rest of the world to receive anticoagulant therapy after VTE diagnosis. In addition, a lower proportion of Asian patients remained on anticoagulant therapy which in part is possibly related to concerns for bleeding complications and the perception that Asians have a lower risk of recurrent VTE.

Based on the dataset derived previously from EINSTEIN-Extension and EINSTEIN-CHOICE in which the one-year cumulative risk of recurrent VTE and major bleeding were estimated according to individual patient baseline risk factor profiles [3], we here present the findings in the subpopulation of Asian patients.

## Methods

### Study design

The randomized and double blind EINSTEIN-Extension and EINSTEIN-CHOICE studies compared the efficacy and safety of once-daily rivaroxaban (20 mg) with placebo and once-daily rivaroxaban (20 mg or 10 mg) with aspirin (100 mg), respectively [4, 5], during extended treatment of VTE. Protocols were approved by the institutional review board at each participating center and written informed consent was obtained from all patients. In a recent prespecified pooled analysis of these studies, the risk of recurrent VTE and major bleeding was estimated according to baseline risk factor profiles [3]. The present study reports the findings of the pooled analysis in the subgroup of Asian patients.

### Patients

The EINSTEIN-Extension and EINSTEIN-CHOICE studies included patients aged 18 years or older who had objectively confirmed, symptomatic proximal DVT or PE and had been treated for 6 to 12 months with an anticoagulant [4, 5]. Patients were excluded from participation if continued anticoagulant therapy was contraindicated or if extended anticoagulant therapy at therapeutic dosages was indicated. For EINSTEIN Choice [5], patients requiring antiplatelet therapy also were ineligible. Additional ineligibility criteria included renal impairment (i.e., calculated creatinine clearance less than 30 ml per minute) or hepatic disease associated with a coagulopathy. For the present subgroup analysis, patients were selected if the investigator had indicated that they were of Asian race.

### Randomization

Using an interactive voice-response system, patients in the EINSTEIN-Extension study were assigned in a 1:1 ratio to receive 20 mg of rivaroxaban or placebo once-daily for 6 or 12 months at the discretion of the local investigator, whereas patients in the EINSTEIN-CHOICE study were assigned, in a 1:1:1 ratio, to receive 20 mg of rivaroxaban, 10 mg of rivaroxaban, or 100 mg of aspirin. Rivaroxaban (20 mg and 10 mg) and matching placebo were provided as identical appearing, immediate release film-coated tablets, whereas aspirin and matching placebo were provided as enteric-coated tablets.

### Risk factor profiles and outcome measures

The definition of the risk factor profiles and outcomes events are reported elsewhere [3]. briefly, the index VTE was centrally graded as provoked by (a) major persistent risk factors (active cancer excluding basal-cell or squamous-cell skin cancer), (b) minor persistent risk factors (inflammatory bowel disease, lower extremity paralysis or paresis, congestive heart failure, body mass index over 30 kg/m^2^, calculated creatinine clearance below 50 ml per minute, family history of venous thromboembolism, or known thrombophilia including deficiency of antithrombin, protein C, or protein S, factor V Leiden or prothrombin gene mutation, and antiphospholipid syndrome), (c) minor transient risk factors (immobilization, travel over 8 h, pregnancy, puerperium, use of estrogen, or lower limb trauma with transient impairment of mobility), or (d) major transient risk factors (major surgery or trauma, or cesarean section). Patients without any of these risk factors were classified as having unprovoked VTE. The primary efficacy outcome was the composite of symptomatic recurrent VTE, VTE-related death, or unexplained death for which PE could not be excluded and the principal safety outcome was major bleeding [4, 5]. 

### Statistical analysis

The analysis included randomized patients administered at least one dose of study medication. Recurrent VTE was considered during the entire study period, whereas major bleeding was considered during the time from administration of the first dose of study drug to 48 h after administration of the last dose. The primary analysis considered the efficacy and safety outcomes in the various risk profile groups using the following hierarchy; unprovoked VTE, provoked VTE with (a) major persistent; (b) minor persistent; (c) minor transient, and (d) major transient risk factors. Cumulative incidences at one year were calculated using the Kaplan-Meier method.

## Results

### Patients

**Fig. 1:**
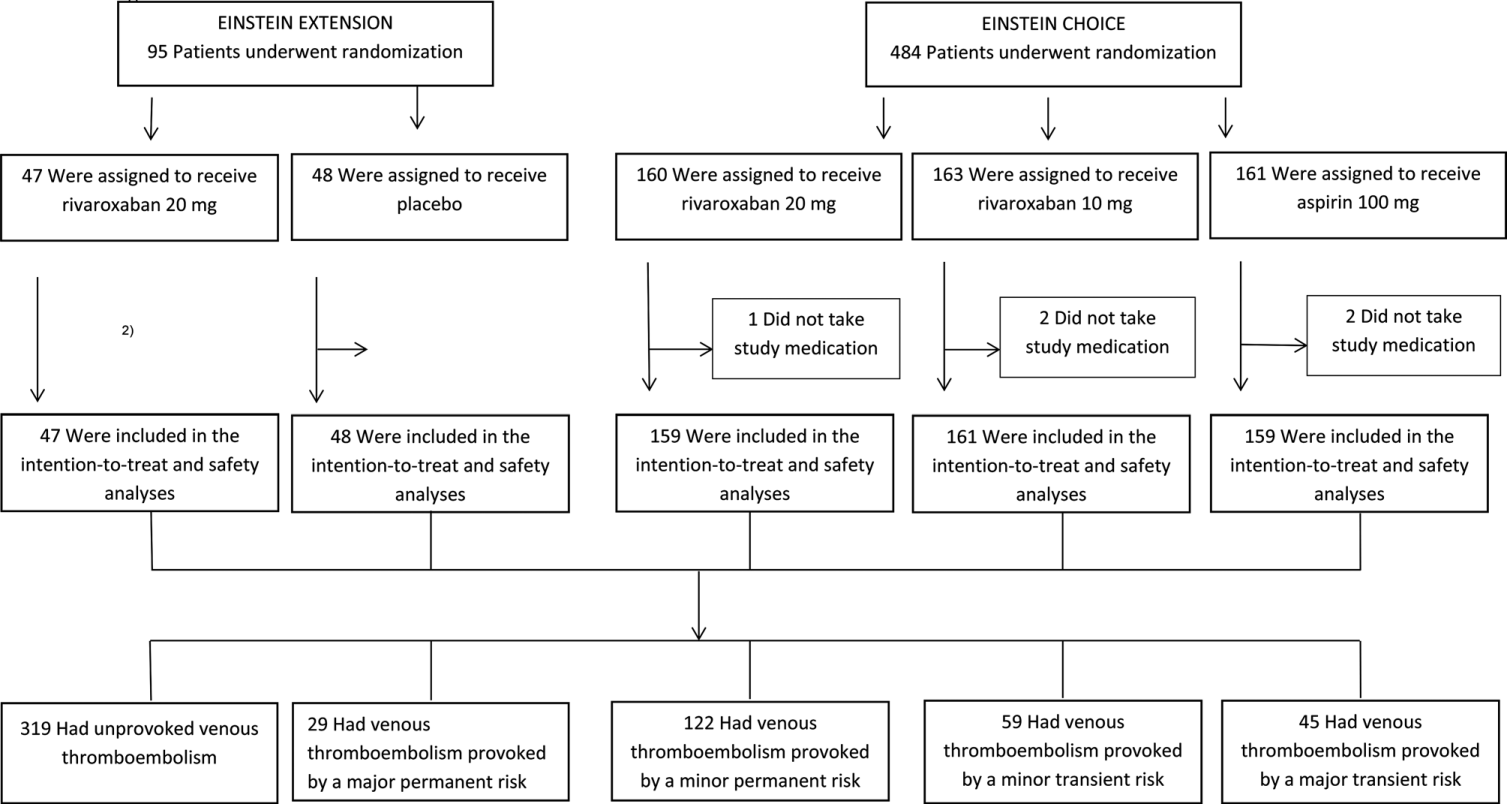
Flow chart

A total of 574 Asian patients were administered study medication and qualified for this analysis: 95 from the EINSTEIN-Extension trial and 479 patients from the EINSTEIN-CHOICE trial. Of these, 367 received rivaroxaban (10 or 20 mg), 159 received aspirin and 48 received placebo (Fig. 1). Demographic and clinical characteristics according to risk profile and treatment assignment are provided in Table 1. Crude incidences of recurrent VTE and major bleeding are provided in Table 2.

**Table 1:** Demographics and clinical characteristics.*

Risk factor profile		EINSTEIN Extension	EINSTEIN CHOICE	EINSTEIN CHOICE	EINSTEIN Extension and EINSTEIN CHOICE	EINSTEIN Extension	EINSTEIN CHOICE
		Rivaroxaban 20 mg	Rivaroxaban 20 mg	Rivaroxaban 10 mg	All Rivaroxaban	Placebo	Aspirin
		*N*=47	*N*=159	*N*=161	*N*=367	*N*=48	*N*=159
Unprovoked	Age, mean– yr	51.1 ±13.4	56.9 ±14.8	57.6 ±14.7	56.4 ± 14.7	43.8 ± 12.5	56.3 ±13.8
	Male sex– no. (%)	17 (58.6)	50 (58.8)	55 (62.5)	122 (60.4)	16 (57.1)	49 (55.1)
	Index DVT only– no. (%)	21 (72.4)	45 (52.9)	41 (46.6)	107 (53.0)	22 (78.6)	43 (48.3)
	Index PE ± DVT– no. (%)	7 (24.1)	40 (47.1)	47 (53.4)	94 (46.5)	1 (3.6)	46 (51.7)
	Duration of previous anticoagulation– no. (%)						
	- < 9 months	19 (65.5)	61 (71.8)	62 (70.5)	142 (70.3)	24 (85.7)	66 (74.2)
	- ≥ 9 months	10 (34.5)	24 (28.2)	26 (29.5)	60 (29.7)	4 (14.3)	23 (25.8)
Provoked by major persistent risk factors	Age, mean– yr	69.0 ±12.7	59.1 ± 6.8	60.0 ± 7.5	60.6 ± 7.8	51.7 ± 7.0	60.7 ±9.7
	Male sex– no. (%)	0	3 (33.3)	2 (33.3)	5 (29.4)	0	2 (22.2)
	Index DVT only– no. (%)	2 (100)	4 (44.4)	3 (50.0)	9 (52.9)	1 (33.3)	5 (55.6)
	Index PE ± DVT– no. (%)	0	5 (55.6)	3 (50.0)	8 (47.1)	2 (66.7)	4 (44.4)
	Duration of previous anticoagulation– no. (%)						
	- < 9 months	1 (50.0)	9 (100)	5 (83.3)	15 (88.2)	2 (66.7)	7 (77.8)
	- ≥ 9 months	1 (50.0)	0	1 (16.7)	2 (11.8)	1 (33.3)	2 (22.2)
Provoked by minor persistent risk factors	Age, mean– yr	56.8 ± 22.8	59.1 ± 19.3	57.8 ± 18.0	58.2 ± 19.0	66.3 ± 16.0	64.9 ± 19.7
	Male sex– no. (%)	5 (45.5)	19 (61.3)	16 (50.0)	40 (54.1)	4 (33.3)	16 (44.4)
	Index DVT only– no. (%)	7 (63.6)	11 (35.5)	11 (34.4)	29 (39.2)	11 (91.7)	18 (50.0)
	Index PE ± DVT– no. (%)	3 (27.3)	20 (64.5)	21 (656)	44 (59.5)	1 (8.3)	18 (50.0)
	Duration of previous anticoagulation– no. (%)						
	- < 9 months	7 (63.6)	28 (90.3)	25 (78.1)	60 (81.1)	8 (66.7)	28(77.8)
	- ≥ 9 months	4 (36.4)	3 (9.7)	7 (21.9)	14 (18.9)	4 (33.3)	8 (22.2)
Provoked by minor transient risk factors	Age, mean– yr	42.8 ± 21.5	47.6 ± 14.7	45.8 ± 15.7	46.2 ± 15.5	47.3 ± 11.4	49.2 ± 15.7
	Male sex– no. (%)	2 (50.0)	5 (33.3)	8 (42.1)	15 (39.5)	0	8 (44.4)
	Index DVT only– no. (%)	3 (75.0)	8 (53.3)	12 (63.2)	23 (60.5)	1 (33.3)	14 (77.8)
	Index PE ± DVT– no. (%)	0	7 (46.7)	7 (36.8)	14 (36.8)	2 (66.7)	4 (22.2)
	Duration of previous anticoagulation– no. (%)						
	- < 9 months	3 (75.0)	10 (66.7)	12 (63.2)	25 (65.8)	3 (100)	13 (72.2)
	- ≥ 9 months	1 (25.0)	5 (33.3)	7 (36.8)	13 (34.2)	0	5 (27.8)
Provoked by major transient risk factors	Age, mean– yr	66.0	50.6 ± 13.2	58.5 ± 14.0	54.6 ± 13.9	66.0 ± 19.8	56.0 ± 13.4
	Male sex– no. (%)	0	8 (42.1)	11 (68.8)	19 (52.8)	2 (100)	5 (71.4)
	Index DVT only– no. (%)	0	12 (63.2)	10 (62.5)	22 (61.1)	1 (50.0)	4 (57.1)
	Index PE ± DVT– no. (%)	1 (100)	7 (36.8)	5 (31.3)	13 (36.1)	1 (50.0)	3 (42.9)
	Duration of previous anticoagulation– no. (%)						
	- < 9 months	0	15 (78.9)	12 (75.0)	27 (75.0)	2 (100)	6 (85.7)
	- ≥ 9 months	1 (100)	4 (21.1)	4 (25.0)	9 (25.0)	0	1 (14.3)

*Plus-minus values are means ± SD.

### Patients with unprovoked VTE

In patients with unprovoked VTE, one-year cumulative incidences of recurrent VTE were 1.6%, 5.8% and 14.8% with rivaroxaban, aspirin, and placebo, respectively. The differences in cumulative incidences in favor of rivaroxaban were 4.1% (95% confidence interval [CI], -1.1-9.3%) versus aspirin and 13.1% (95% CI, -0.4-26.7%) versus placebo (Table 2). Major bleeding in patients with unprovoked VTE occurred in 2 patients (one-year cumulative incidence, 1.2%) who received rivaroxaban, and in none who received aspirin or placebo, for a difference of 1.2% (95% CI, -0.5-2.8%) in favor of aspirin and placebo.

### Patients with persistent risk factors

One-year cumulative incidences of recurrent VTE in patients with minor persistent risk factors were 0%, 9.3%, and 0% in those who received rivaroxaban, aspirin, and placebo, respectively, for a difference in favor of rivaroxaban of 9.3% (95% CI, -0.8-19.4%) versus aspirin and 0% versus placebo. One-year cumulative incidences of major bleeding in patients with VTE provoked by a minor persistent risk factor were 1.5% in rivaroxaban recipients and 0% in those who received aspirin or placebo.

Only 29 patients with major persistent risk factors were included and none had recurrent VTE or a major bleeding event (Table 2).

**Table 2:** Crude incidences of recurrent venous thromboembolism and major bleeding in Asian patients receiving rivaroxaban, placebo, or aspirin according to baseline risk factor profiles

Risk factor profile		EINSTEIN Extension	EINSTEIN CHOICE	EINSTEIN CHOICE	EINSTEIN Extension and EINSTEIN CHOICE	EINSTEIN Extension	EINSTEIN CHOICE
		Rivaroxaban 20 mg	Rivaroxaban 20 mg	Rivaroxaban 10 mg	All Rivaroxaban	Placebo	Aspirin
		*n*=47	*n*=159	*n*=161	*n*=367	*n*=48	*n*=159
All patients, N (%)							
Recurrent VTE		0	1 (0.6)	2 (1.2)	3 (0.8)	4 (8.3)	8 (5.0)
Major bleeding		0	3 (1.9)	2 (1.2)	5 (1.4)	0	0
Unprovoked, N (%)		29 (61.7)	85 (53.5)	88 (54.7)	202 (55.0)	28 (58.3)	89 (56.0)
Recurrent VTE		0	1 (1.2)	2 (2.3)	3 (1.5)	4 (14.3)	5 (5.6)
Major bleeding		0	2 (2.4)	0	2 (1.0)	0	0
Provoked	Major persistent risk factors, N (%)	2 (4.3)	9 (5.7)	6 (3.7)	17 (4.6)	3 (6.3)	9 (5.7)
	Recurrent VTE	0	0	0	0	0	0
	Major bleeding	0	0	0	0	0	0
	Minor persistent risk factors, N %)	11 (23.4)	31 (19.5)	32 (19.9)	74 (20.2)	12 (25.0)	36 (22.6)
	Recurrent VTE	0	0	0	0	0	3 (8.3)
	Major bleeding	0	1 (3.2)	0	1 (1.4)	0	0
	Minor transient risk factors, N (%)	4 (8.5)	15 (9.4)	19 (11.8)	38 (10.4)	3 (6.3)	18 (11.3)
	Recurrent VTE	0	0	0	0	0	0
	Major bleeding	0	0	1 (5.3)	1 (2.6)	0	0
	Major transient risk factors, N (%)	1 (2.1)	19 (11.9)	16 (9.9)	36 (9.8)	2 (4.2)	7 (4.4)
	Recurrent VTE	0	0	0	0	0	0
	Major bleeding	0	0	1 (6.3)	1 (2.8)	0	0

VTE denotes venous thromboembolism

### Patients with transient risk factors

Recurrent VTE in patients with minor transient risk factors occurred in none of the 38, 18, and 3 patients who received rivaroxaban, aspirin and placebo, respectively, whereas major bleeding occurred in 1 patient (one-year cumulative incidence, 2.9%) who received rivaroxaban and in none who received aspirin or placebo.

Recurrent VTE in patients with major transient risk factors occurred in none of the 36, 2, and 7 patients who received rivaroxaban, aspirin and placebo, respectively, whereas major bleeding occurred in 1 patient (one-year cumulative incidence, 2.9%) who received rivaroxaban and none who received aspirin or placebo.

## Discussion

Our post-hoc analysis shows that Asian patients with VTE administered extended treatment with rivaroxaban, aspirin or placebo have low incidences of recurrent VTE and major bleeding which are similar to those observed in patients in the rest of the world [4, 5]. However, the number of Asian patients included in our analysis was too low to provide firm evidence of the efficacy of rivaroxaban for patients presenting with major or minor provoked transient or permanent risk factors. In Asian patients with unprovoked VTE, differences between rivaroxaban and aspirin or placebo in one-year incidences of recurrent VTE (i.e., 4.1% and 13.1%, respectively) were similar to those observed in the entire cohort of patients (i.e., 3.9% and 8.0%, respectively) on which this substudy is based [3]. Rivaroxaban was associated with a major bleeding rate similar to those with aspirin or placebo. The high risk of recurrent VTE, if anticoagulation is stopped in Asian patients with unprovoked VTE, justify current guidelines that suggest that patients with unprovoked VTE may benefit from extended anticoagulation. Therefore, with its fixed-dose regimen which does not require laboratory monitoring and the option to lower the dose to 10 mg once-daily, rivaroxaban is an attractive option for extended treatment of VTE also in Asian patients.

Our observations in Asian patients with unprovoked VTE are also consistent with those made in a recent systematic review and network meta-analysis of extended treatment of venous thromboembolism [7]. The results of this systemic review, which involved a large number of patients, showed that therapy with standard-intensity vitamin K antagonist (VKA), full-dose direct oral anticoagulants (DOAC) and reduced-dose DOAC are associated with a lower risk of recurrent VTE, while only standard-intensity VKA therapy was associated with a higher risk of major bleeding. Unfortunately, only relatively small subgroups of Asian patients could be evaluated in our analysis. Consequently, it was not possible to corroborate in the Asian subpopulation our earlier observations of fewer recurrent VTE events with (reduced-dose) anticoagulation in those presenting with provoked persistent or transient risk factors. However, in patients with VTE and a major persistent risk factor (i.e., cancer), the increased risk of recurrent VTE is widely recognized and extended anticoagulation is generally recommended, albeit individualized based on the patient’s bleeding risk, type of cancer, potential for drug–drug interactions, as well as patient and clinician preferences [8]. Conversely, in patients with VTE provoked by a major transient risk factor (such as major surgery or trauma) the risk of recurrent VTE is now widely considered to be too low to justify extended anticoagulation, provided that the risk factors have resolved [3, 9 and 10]. However, in patients with VTE provoked by a minor persistent or transient risk factors, the recent novel finding of an increased risk of recurrent VTE [3], if anticoagulation therapy is stopped, requires additional studies since the risk of recurrent VTE is potentially not dissimilar to the risk associated with unprovoked VTE and these patients commonly receive shorter-duration therapy [3, 11].

## Conclusions

This post-hoc subgroup analysis in Asian patients with VTE shows that those presenting with unprovoked VTE have similar incidence of recurrent VTE and major bleeding as compared to non-Asian patients and are likely to benefit from extended anticoagulation with rivaroxaban. Subgroups of Asian patients with VTE provoked by persistent or transient risk factors were too small to corroborate the observations made in non-Asian patients. With the option of two-dose rivaroxaban regimens, selection can be personalized based on individual benefit–risk considerations.

## Data Availability

All data generated or analyzed during this study are included in this manuscript.

## Abbreviations

VTE: Venous thromboembolism
VKA: Vitamin K antagonist
DOAC: Direct oral anticoagulant
